# Fungal Communities in Asymptomatic and Symptomatic Needles of *Pinus* spp. Affected by Pine Needle Diseases

**DOI:** 10.3390/microorganisms14010088

**Published:** 2025-12-31

**Authors:** Nebai Mesanza, Jenny Aitken, Amelia Uria, Eugenia Iturritxa

**Affiliations:** 1 Department of Forest Science, Neiker-BRTA, Instituto Vasco de Investigación y Desarrollo Agrario, Granja Modelo s/n, Antigua Carretera Nacional 1, Km. 355, 01192 Arkaute, Spain; 2 Jenny Aitken Biotechnologies Limited, P.O. Box 11236, Papamoa 3151, New Zealand; jenny_aitken@xtra.co.nz; 3 Euskadiko Basogintza Elkarteen Konfederakundea, Dionisio Aldama, 7 1ºB, 01470 Amurrio, Spain; araba@basoa.org

**Keywords:** fungal communities in needles, symptomatic and asymptomatic needles, *Lophodermium*, *Pinus*, metabarcoding, needle cast, needle blight

## Abstract

The aim of this study was to determine fungal diversity and composition in an area of high host diversity and identify the organisms involved in the appearance of symptoms in *Pinus* needles. Asymptomatic and symptomatic live needle samples were obtained from different *Pinus* spp. in an arboretum with confirmed presence of brown spot needle blight. The samples were analysed using high-throughput sequencing of fungal ITS2rDNA. Ascomycota dominated all samples, with *Lophodermium* as the most abundant genus, although it showed lower representation in symptomatic needles. Other genera with recognised pathogenic potential, including *Lecanosticta*, *Pestalotiopsis*, *Cyclaneusma*, *Rhizosphaera*, *Neophysalospora*, and *Cenangium*, were also detected, whereas the *Dothistroma* genus was absent despite its presence in the region. Alpha diversity was higher in asymptomatic needles, with a significant difference only for the Shannon index, while Bray–Curtis dissimilarity revealed significant shifts in community composition between needle types. Functional guilds were dominated by pathotroph–saprotroph trophic mode, and the functional guild ‘plant pathogen’ was the most abundant across samples. These findings identify fungal genera associated with symptomatic and asymptomatic needles and provide guidance for future targeted isolation and detailed morphological and molecular identification using more resolutive techniques, enabling a deeper understanding of pathogenic community presence and their potential synergistic interactions.

## 1. Introduction

The frequency of needle disease outbreaks and their severity in *Pinus* L. spp. have increased over recent decades, likely triggered by climate change and cultural practices such as establishing large plantations of susceptible host species. Needle diseases may result in repeated premature needle loss, which gradually weakens the host, reduces timber yield, and leads to tree death in some cases [1,2,3]. The most significant needle pathogens in *Pinus* spp. are *Dothistroma* Hulbary spp., *Lecanosticta acicola* (Thüm.) Syd., *Lophodermium* Chevall. spp., *Lophodermella* Höhn., *Phytophthora* de Bary spp., and *Cyclaneusma minus* (Butin) DiCosmo, Peredo and Minter [4,5,6,7]. Among these, *Dothistroma* spp. are the most destructive pine foliar pathogens [8]. Their similar symptomatology has been associated with various needle diseases such as Dothistroma needle blight, brown-spot needle blight, *Lophodermium* needle cast, and *Cyclaneusma* needle cast that affect several conifer species [9].

Brown spot needle blight caused by *L. acicola* is one of the most important diseases in the Basque Country, where forestry is principally based on monospecific stands of *Pinus radiata*. The earliest record of *L. acicola* in Europe dates to 1942, when the pathogen was reported causing defoliation in the Basque Country [10]. At that time, it was observed exclusively on *P. radiata* D. Don grown under suboptimal conditions, and not on other locally assessed *Pinus* species [11]. Prior to 2008, disease outbreaks were largely confined to humid environments—particularly valley bottoms and high-density plantations—despite the widespread cultivation of *P. radiata* in the region. In subsequent years, the distribution and severity of *L. acicola* have increased markedly. Recent surveys have documented *L. acicola* infecting multiple *Pinus* species—including *P. radiata*, *P. nigra* Arnold, *P. sylvestris* L., *P. ponderosa* Douglas ex C. Lawson, *P. elliottii* Engelm., and *P. brutia* Ten.—highlighting the pathogen’s broad host range and colonisation versatility [12,13]. Furthermore, analyses using simple sequence repeat markers and whole-genome sequencing showed that this pathogen exhibited high genetic diversity in the region and confirmed the presence of northern and southern lineages of *L. acicola* [14,15]. The presence of its sexual form was also confirmed in the region [16].

Environmental and host-related factors determine the differences in fungal community composition, diversity, and richness [17,18]. Fungal interactions with their hosts depend on plant compounds, morphology, and life-history traits. Phylogenetically related host species show association with their mycobiomes [18,19,20,21]. This association or host specialisation may be stronger for pathogenic than for non-pathogenic fungi [18]. Additionally, several needle fungal pathogens spend part of their life cycle as endophytes or epiphytes without causing apparent damage, and visible infection is triggered under certain environmental conditions or when the hosts are weakened by stress [22,23,24,25,26,27].

In the absence of other feasible management methods, the selection of tolerant hosts such as resistant host species, subspecies, or hybrids is recommended for controlling the spread and impact of a pathogen [28]. However, adequate caution must be exercised when complexes of pathogenic fungi exist in a region because a host tolerant to a specific pathogen may still be susceptible to the other pathogens. The susceptibility of large plantations of non-native tree species to pathogens is associated with inadequate species/site matching, the use of individuals with a limited genetic base, and heavy reliance on one or two species in plantation strategies for a region [29]. Additionally, newly established exotic tree species may undergo a pest-free period of variable duration because of the absence of co-evolved pathogens in the area and the inability of native pathogens to adapt to the new hosts [29,30]. Thus, building resilient forest ecosystems requires more effort than the mere introduction of new tree provenances or species. Consequently, a high level of host genetic diversity is a key requirement in both plantations and native forests to assimilate and adapt to adverse conditions such as climate change and diseases [31].

Plants in arboreta and botanical gardens (sentinel species) are important resources for detecting newly introduced pathogens and identifying potential risks resulting from novel pest–host interactions [32] or native pest–exotic forest species interactions. However, arboreta usually contain representative tree species in small numbers; hence, the issues related to extensive plantations may go unnoticed [33]. Nevertheless, the results from arboreta regarding the suitability of new exotic tree species for a region may be useful, albeit preliminary. This is especially true if the experimental sites are located in areas of high exposure to pathogens and representative of the environmental conditions of the region [33].

This study aimed to characterise the fungal diversity and composition in symptomatic and asymptomatic needles of *Pinus* spp. using high-throughput sequencing of the ITS2 region. We hypothesised that symptomatic needles would host distinct fungal community structures, including higher representation of genera with pathogenic potential, and expected that these differences would help identify fungal genera of interest for future isolation and high-resolution morphological and molecular identification.

## 2. Materials and Methods

### 2.1. Study Site and Sampling

The study site was located in the Umbemendi2 arboretum (AR19) (43°21′05.3″ N, 2°55′47.4″ W) at Laukiz, Biscay, Basque Country, Spain. This arboretum was planted between 2011 and 2013 on a harvested *P. radiata* plantation as part of the European project REINFFORCE (https://www.efi.int/projects/reinfforce-resource-infrastructures-monitoring-adapting-and-protecting-european-atlantic) (accessed on 26 December 2025). During establishment, the trees were labelled with IDs to facilitate identification. The arboretum is currently surrounded by plantations of *P. radiata*, *P. nigra*, and *Eucalyptus* L’Hér. spp., and native deciduous forest that primarily comprises the *Quercus* genus. The stand is north-oriented with a 5% slope and cambisol soil. The mean annual temperature of the area is 14 °C, with a mean of 6.2 °C in the coldest month, and the average annual precipitation is 1233 mm.

Samples were collected from nine *Pinus* species. Of these, *P. brutia* Ten., *P. elliottii* Engelm., *P. nigra* Arnold, *P. pinaster* Ait., *P. pinea* L., *P. ponderosa* Douglas ex C. Lawson, *P. sylvestris* L., and *P. taeda* L. were planted in the arboretum and were 12 years old at the time of sample collection. The *P. radiata* D. Don. trees were located at the border of the arboretum because the *P. radiata* species was not chosen as part of the REINFORCE arboreta. The selected *P. radiata* were approximately 15 years old during sampling. Two groups of needle samples were collected from the lower parts of five trees for each *Pinus* species. One group comprised needles showing symptoms of fungal defoliators (discolouration, spots, bands, or dead tips), and the other group comprised asymptomatic needles. Samples from the same *Pinus* species and group (asymptomatic or symptomatic) were combined and stored at 4 °C in paper envelopes overnight before DNA extraction. The needles were cut with sterilised scissors into approximately 0.5 cm pieces, and the needle tissue disruption was performed in a Qiagen Tissuelyser II (QIAGEN GmbH, Hilden, Germany) with sterile metal beads (Ø 2.5 mm). DNA was extracted from approximately 100 mg of the needle powder using the innuPREP Plant DNA Mini Kit (Analytik Jena AG, Jena, Germany).

### 2.2. Illumina Library Construction, Sequencing, and Data Processing

The internal transcribed spacer 2 (ITS2) region of the nuclear ribosomal DNA was used as a DNA barcoding marker for the molecular identification of fungal taxa using the universal primers ITS86 (F): GTGAATCATCGAATCTTTGAA [34] and ITS4 (R): TCCTCCGCTTATTGATATGC [35], both of which contain Illumina adapter sequences. The PCR cycle conditions were: 5 min at 95 °C; 40 cycles of 30 s at 94 °C, 30 s at 55 °C, and 72 °C for 1 min; and final extension for 7 min at 72 °C. Each PCR reaction contained 25 µL of Master Mix Supreme NZYTaq II 2X colourless (NZYTech, Lisbon, Portugal), 0.5 µM primers, and 2 µL of DNA at a final volume of 50 µL. PCR amplification was performed in triplicate for each sample.

Library construction, DNA amplification, assemblage of paired-end reads, filtering of bad quality sequences, and sequence clustering of operational taxonomic units (OTUs) were performed by Macrogen, Inc. Library construction and DNA amplification were performed using the Herculase II Fusion DNA Polymerase Nextera XT Index Kit V2 by following the Illumina 16S Metagenomic Sequencing Library Preparation Protocol (Part #15044223, Rev. B). Paired-end sequencing (2 × 301 bp) was performed using the MiSeq platform (Illumina, San Diego, CA, USA).

Paired-end reads from each sample were assembled using FLASH 1.2.11 [36] with default parameters. Reads were filtered with a quality score offset of 33, and low-quality, ambiguous, and chimeric sequences were removed. The remaining sequences were clustered into OTUs at a 97% similarity threshold using the CD-HIT-OTU software v.4.8.1 [37].

To classify the representative sequences in QIIME2 2024.2 [38], a Naïve Bayes classifier was trained using the “qiime feature-classifier fit-classifier-naive-bayes” command with the UNITE all eukaryotes developers dataset v10 (04.04.2024) [39], which included singletons set as RefS (in dynamic files) using the “qiime feature-classifier classify-sklearn” command [40]. Unclassified OTUs and those not belonging to the kingdom Fungi, such as Viridiplantae, Rhizaria, Alveolata, and Eukaryota_kingdom_Incertae_sedis, were removed from the dataset using the “qiime taxa filter-table” command.

Technical replicates were merged in the dataset by mean (with qiime feature-table group command and parameter “mode” set as mean-ceiling), and features with fewer than 10 counts were removed using “qiime feature-table filter-features” to minimise the inflation of rare OTUs in community analysis [41,42].

Fungal OTUs were assigned to trophic modes and guilds using the FUNGuild.py Python script v1.0 [43]. Assignments were retained only if they reached the ‘Probable’ or ‘Highly Probable’ confidence ranking. Trophic modes were defined as follows: pathotrophs obtain nutrients by harming host cells (including phagotrophs); symbiotrophs receive nutrients by exchanging resources with host cells; and saprotrophs acquire nutrients by breaking down dead host cells [43]. Functional guilds were constrained to the following categories: undefined saprotrophs, plant saprotrophs, and plant pathogens.

### 2.3. Community Analysis

Community analysis was performed using QIIME 2 2024.2 [38]. Rarefaction curve analysis was performed on the dataset (via the “qiime diversity alpha-rarefaction” command), and the OTU table was rarefied to the smallest library size (sequence depth of 93,316) via the “qiime feature-table rarefy” command for downstream community diversity analysis to minimise the biases associated with different sample sizes. Alpha diversity was calculated using observed OTUs, Shannon’s index, and Simpson’s index (1-D) (with the “qiime diversity alpha” command). Significant differences were determined using the Kruskal–Wallis test (*p* < 0.05) (with the “qiime diversity alpha-group-significance” command). Beta diversity was calculated using the Bray–Curtis dissimilarity index and Jaccard similarity index (by the “qiime diversity core-metrics” command). Permutational multivariate analysis of variance (PERMANOVA) [44] was used to test the association between fungal beta diversity and needle status (via the “qiime diversity beta-group-significance” command). For significant PERMANOVA results, differences in community dispersion were checked using PERMDISP [45] because PERMANOVA results may be affected by beta dispersion. Data of the Bray–Curtis dissimilarity and Jaccard similarity distance matrices generated in QIIME2 were used to perform principal coordinate analysis (PCoA) in R-4.4.2. PCoA images were generated with tidyverse [46] and qiime2R v0.99 [47]. https://github.com/jbisanz/qiime2R packages (accessed on 26 December 2025).

Analysis of microbiome compositions with bias correction (ANCOMBC) [48] was applied to identify taxa that were differentially abundant between asymptomatic and symptomatic needles at the genus and family levels using the “qiime composition ancombc” command. Owing to the small sample size, a conservative variance estimator was used for the test statistics. OTUs with Holm-adjusted *p* < 0.05 were considered significant. Graphic representations of ANCOMBC results were obtained using the “qiime composition da-barplot” command.

## 3. Results

### 3.1. Sequencing Data and Relative Abundance of Fungal OTUs

High-throughput sequencing of the ITS2 region of the nuclear ribosomal DNA yielded a total of 9,432,191 reads with a mean of 184,945 reads per sample. After quality evaluation, 7,012,105 clean reads were obtained with a mean of 137,492 ± 16,352 reads per sample at a range of 106,871–176,920 reads. In total, 3227 OTUs were generated. Non-fungal and low-frequency OTUs were filtered to yield 1338 OTUs.

**Figure 1:**
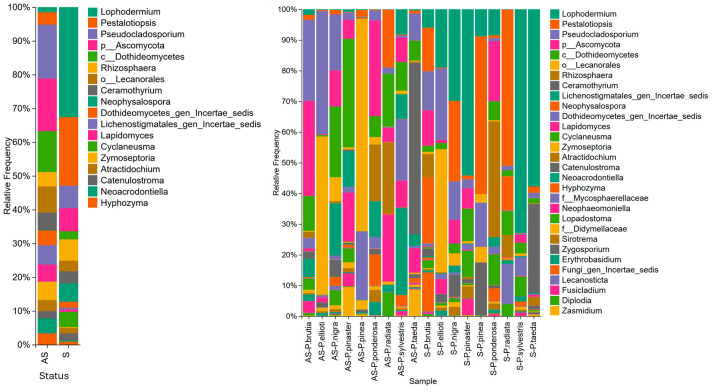
(**Left**): Relative abundance of fungal genera by sample status: asymptomatic (AS) and symptomatic (S). (**Right**): Relative abundance of fungal genera by sample status: asymptomatic (AS), symptomatic (S), and pine species.

The majority (87%) of fungal OTUs were assigned to Ascomycota, and 11% were assigned to Basidiomycota. The most abundant OTUs belonged to the family Rhytismataceae. The genus *Lophodermium* showed the highest presence and accounted for 22.2% of the OTUs in symptomatic needles and 0.9% in asymptomatic needles (Figure 1).

OTUs classified as *Pestalotiopsis* sp. were highly abundant in symptomatic samples (13.34%) than in asymptomatic samples (2.01%). They were abundant in symptomatic samples of *P. pinea* (37.7%), *P. nigra* (21.1%), *P. brutia* (12.2%), and *P. radiata* (42%). Additionally, *Rhizosphaera* was abundant (2.3% in asymptomatic and 4.1% in symptomatic samples) in symptomatic (31.8%) and asymptomatic (15.8%) *P. ponderosa* samples and symptomatic (6.6%) and asymptomatic (1.4%) *P. brutia* samples (Figure 1).

*Neophysalospora* was more abundant in symptomatic samples (3.5%) than in asymptomatic samples (0.04%) and was primarily detected in high abundance in symptomatic needles of *P. pinea* (1% of OTUs), *P. brutia* (18% of OTUs), and *P. radiata* (9% of OTUs). The relative abundance of *Cyclaneusma* was higher in symptomatic (3%) than in asymptomatic needles (0.03%), with higher relative abundances in symptomatic needles of *P. pinaster* (6.1% of OTUs), *P. sylvestris* (4.7% of OTUs), *P. ponderosa* (8.4% of OTUs), and *P. radiata* (7.6% of OTUs) (Figure 1).

**Figure 2:**
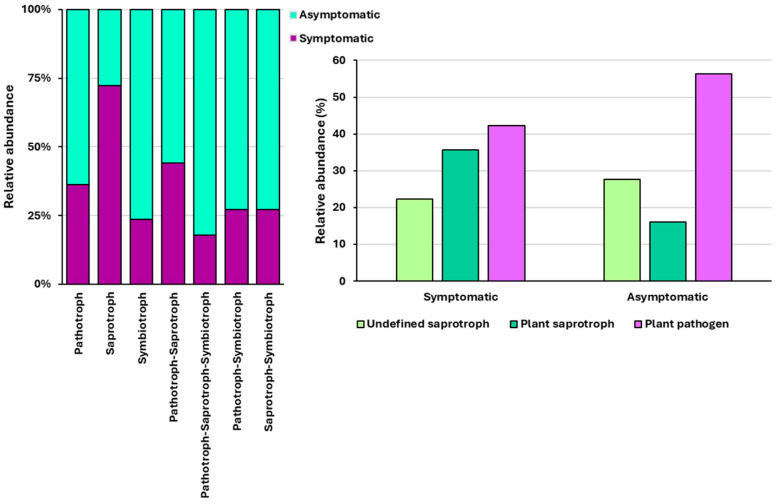
Relative abundance of identified trophic modes (**left**) and functional guilds (**right**) in asymptomatic and symptomatic needles.

Trophic modes and functional guilds were successfully assigned to 752 OTUs (77.3%) using FUNGuild. The remaining OTUs were unassigned because they were not identified at the genus level. In both asymptomatic and symptomatic needles, trophic mode pathotroph–saprotroph represented the largest proportion (40.5%), followed by saprotroph (36.7%) and pathotroph (12%). When comparing asymptomatic and symptomatic needles, all trophic modes were higher in asymptomatic needles, except for the saprotroph mode. Plant pathogens were the most abundant in both symptomatic and asymptomatic needles, whereas plant saprotrophs were the least abundant in asymptomatic needles and undefined saprotrophs in symptomatic needles (Figure 2).

### 3.2. Community Analysis

#### 3.2.1. Rarefaction Curves and Diversity Analysis

Rarefaction curves generated by plotting Shannon and Simpson diversity metrics against sequencing depth levelled off in all samples before reaching the sequence depth chosen to rarefy the OTU table for diversity analysis. This finding indicated that fungal diversity was adequately represented. In contrast, the rarefaction curves for the observed OTUs did not reach a plateau in any of the samples, which indicated that rare species may not have been adequately represented.

**Figure 3:**
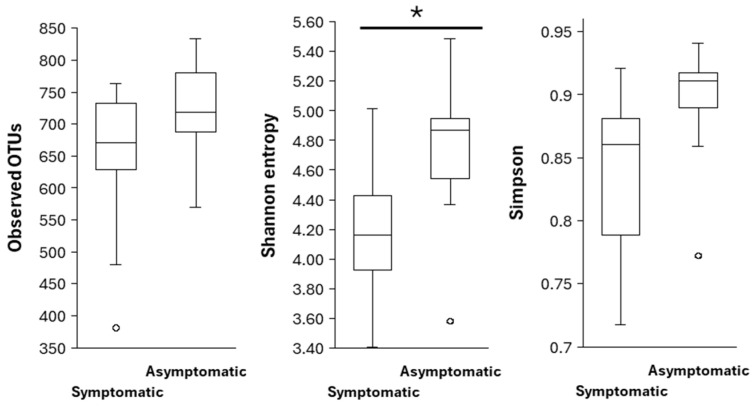
Alpha diversity indices in symptomatic and asymptomatic *Pinus* spp. needles. (*) indicates a significant difference (*p* < 0.05) based on the Kruskal–Wallis test. **Left**: Observed OTUs; **Middle**: Shannon entropy; **Right**: Simpson.

After data rarefaction (at sampling depth of 93,316), a mean of 608 observed OTUs per sample was obtained, ranging from 381 to 780. The highest number of OTUs was detected in asymptomatic needles of *P. brutia*, and the lowest in symptomatic needles of *P. pinea*. However, the difference in OTUs between the asymptomatic and symptomatic needles of *Pinus* spp. was not significant (H = 1.76; *p* = 0.18). The Shannon diversity index, which emphasises richness, suggested that fungal communities from symptomatic needles were significantly less diverse than those from asymptomatic needles (H = 4.31; *p* = 0.038). The Simpson diversity index, which emphasises evenness, indicated no significant difference between the fungal communities in the asymptomatic and symptomatic needles (H = 2.96; *p* = 0.085) (Figure 3).

**Figure 4:**
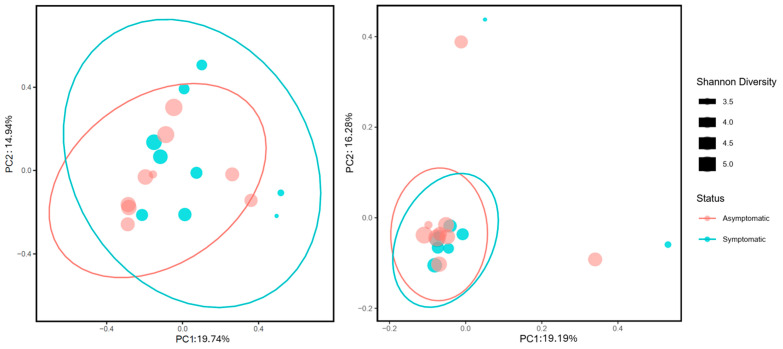
Principal coordinate analysis (PCoA) of fungal communities associated with asymptomatic (red) and symptomatic (blue) needles of *Pinus* spp. based on beta diversity metrics: Bray–Curtis (**left**) and Jaccard (**right**). Confidence ellipses were calculated at 95%. Circle size indicates the Shannon diversity index. Values of the Shannon diversity index correspond to the size of the black squares present in the right.

With respect to beta-diversity metrics, the PERMANOVA result for Bray–Curtis dissimilarity (fraction of overabundant counts) was significant (pseudo-F = 1.748; *p* = 0.032), whereas the Jaccard similarity index (fraction of unique features regardless of abundance) was not significant (pseudo-F = 0.781; *p* = 0.793) (Figure 4). Analysis of beta dispersion (PERMDISP) was not significant for Bray–Curtis distances (*p* = 0.7), which indicated that the significant differences obtained through PERMANOVA were due to differences between symptomatic and asymptomatic needle fungal communities and not because of differences in dispersion within the groups.

#### 3.2.2. Differential Abundance Analysis of Taxa

**Figure 5:**
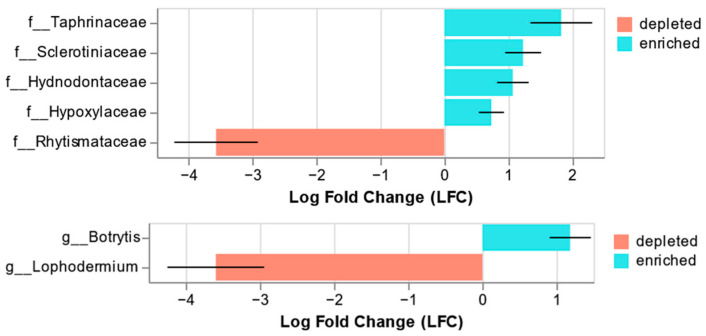
Significant results from the ANCOM-BC. Significant differences in relative abundance (q < 0.05) at the family (**top**) and genus (**bottom**) levels are shown. Negative LFC values indicate a decrease in the relative abundance of fungal taxa in asymptomatic needles compared with that in symptomatic needles, whereas positive values indicate an increase.

Analysis of microbiome composition with bias correction (ANCOMBC) showed significant differences between organisms in asymptomatic and symptomatic needles at the fungal genus and family levels (Figure 5). At the family level, Rhytismataceae was significantly depleted in asymptomatic needles (q = 0.00001), and Taphrinaceae (q = 0.04), Sclerotiniaceae (q = 0.003), Hydnodontaceae (q = 0.004), and Hypoxylaceae (q = 0.046) were significantly enriched. At the genus level, *Lophodermium* was significantly depleted in asymptomatic needles (q = 0.00002), whereas *Botrytis* was significantly enriched (q = 0.01).

## 4. Discussion

We performed high-throughput sequencing of the ITS region of fungal rDNA and compared the fungal communities of symptomatic and asymptomatic needles collected from different *Pinus* species at an arboretum in Biscay, northern Spain, that was severely affected by pine needle blight.

Ascomycota was identified as the predominant phylum in the analysed needles, and this trend was observed in other conifer mycobiome studies [49,50,51,52,53]. The most abundant pathogens belonged to the family Rhytismataceae. Several species in this family are considered potential primary or opportunistic pathogens of conifers, especially in *Pinus* spp. Depending on their pathogenic capacity, they cause premature needle loss (needle cast) or sudden foliar death (needle blight) [25,54]. *Lophodermium* was the most abundant fungal genus, especially in symptomatic needles, and was the only fungal genus that was significantly depleted in asymptomatic needles. *Lophodermium* species are considered weak pathogens, with the exception of *L. seditiosum*, which causes severe damage to nurseries and plantations [25]. Additionally, *L. pinastri* and *L. conigenum* belong to the *Lophodermium* genus*. L. pinastri* is a non-pathogenic endophyte that actively develops at the beginning of needle senescence and damages the weakened or dead needles [25,50]. However, a *P. radiata* selection trial in Tasmania showed that this species was the only one that correlated with high levels of needle cast [55]. Generally, *L. conigenum* is considered a weak pathogen, although it has been associated with disease symptoms in *P. mugo* and *P. taeda* [56,57]. Additionally, it is often present in New Zealand *P. radiata* trees previously affected by *Cyclaneusma minus*. Furthermore, the impact of certain species is worse in nurseries than in plantations [54].

*Pestalotiopsis* spp. were detected in all analysed samples. They showed high relative abundance, especially in symptomatic needles of *P. pinea*, *P. nigra*, *P. brutia*, and *P. radiata*. The significance of *Pestalotiopsis* species is increasing in temperate forests. They usually act as secondary pathogens that cause severe damage to different plant structures [50]. Furthermore, they are associated with the decline of *P. pinea* and considered a potential threat to the health of pine forests in the Mediterranean Basin [26]. *Cyclaneusma* was present in all samples except in symptomatic needles of *P. pinea*. This pathogen is distributed worldwide; however, it is particularly damaging to *P. radiata* plantations [27]. Its pathogenicity is highly variable and strongly dependent on the host genotype [58]. *Rhizosphaera* was identified in all *Pinus* species analysed in this study. *Neophysalospora* was more abundant in symptomatic samples of *P. pinea*, *P. nigra*, and *P. radiata*. This genus contains only one fungal species, *N. eucalypti*, which is a pathogen associated with brown leaf spots in eucalyptus plantations [59]. Previously, *N. eucalypti* was isolated from symptomatic needles of *P. radiata* trees located in Cantabria, Spain [60]. *Lecanosticta* was identified in *P. pinaster*, *P. brutia*, *P. elliotti*, *P. ponderosa*, *P. radiata*, and *P. taeda*; however, only the *P. radiata* needles showed a high relative abundance of this pathogen.

One OTU identified as *Botrytis* was present in all the samples analysed, and it was the only significantly enriched genus in asymptomatic needles. This genus contains approximately 30 species, with *B. cinerea* being the most studied member. This species has shown potential to switch between different lifestyles: facultative endophytic to necrotrophic behaviour. It has been reported as a pathogen in several *Pinus* species. Other species in the *Botrytis* genus show an endophytic lifestyle with facultative necrotrophy or non-pathogenic associations [61]. Thus, further determination of the *Botrytis* species in the study area could clarify its possible role in *Pinus* needles.

Coexistence of pathogens causes pine needle damage [50,56,57,60,62]. Early mutualistic associations may shift toward competitive dynamics, in which the superior species becomes dominant because of the potential differences in nutritional requirements, substrate suitability, production of antifungal compounds, or favourable climatic conditions. This may be attributed to the fact that fungal interactions are highly context-dependent and shaped by factors such as host and pathogen genotype and composition of the surrounding fungal community [50]. Domination of *C. minus* and *L. pinastri* in association with lower fungal diversity was observed in *Pinus sylvestris* symptomatic and asymptomatic needles, and we concluded that *Cyclaneusma* needle cast in Poland may be caused by *C. minus*, accompanied by *C. ferruginosum*, *L. seditiosum*, *L. pinastri*, and *Sydowia polyspora* [50]. White pine needle damage is a disorder in *Pinus strobus* and was principally associated with *Lecanosticta acicola*, in addition to other needle cast fungi, including *Lophophacidium dooksi* and *Bifusella linearis* [62]. In this study, *Lophodermium* and *Pestalotiopsis* were predominant in symptomatic needles, although they were also present in asymptomatic needles. The other fungi associated with symptomatic needles were *Neophysalosphora* and *Cyclaneusma*, although their relative frequency highly varied in the different analysed host species.

Fungal community characteristics were analysed in symptomatic and asymptomatic needles. Alpha diversity indices were higher in asymptomatic needles, although only the Shannon index was statistically significant. Bray–Curtis dissimilarity (fraction of overabundant counts) was significant between symptomatic and asymptomatic needles, whereas the Jaccard similarity index (fraction of unique features regardless of abundance) was not significant. Additionally, the Bray–Curtis index was significant when the structure of endophytic fungal communities was studied in healthy and symptomatic needles of *Pinus sylvestris* var. *mongolica* [46]. This trend towards increased fungal community diversity in asymptomatic needles has been reported previously [49,63]. This may be attributed to the dominance of certain pathogens that outcompete other species or environmental changes that favour pathogenic growth [49]. In contrast, the opposite trend was observed for fungal community richness in *P. taeda* needles, which may be attributed to opportunistic pathogens and saprophytes invading the weakened or dead tissues [57].

In the current study, the trophic mode pathotroph–saprotroph represented the largest proportion in both asymptomatic and symptomatic needles, and the functional guild ‘plant pathogen’ was the most abundant in symptomatic and asymptomatic needles. The high proportion of plant pathogens detected in symptomless needles may be explained by the presence of latent pathogens that remain dormant until favourable conditions arise [49]. Additionally, fungi classified as parasites of fungi were detected. For example, *Tremella* was present in all but one of the analysed samples. Previous studies have found members of this genus parasitising *Lophodermium* spp. [64,65,66].

Environmental DNA metabarcoding is an effective tool for detecting pathogens, including those at dormant or latent stages, and causing no visible symptoms [24,50,53]. Additionally, it enables the detection of slow-growing microorganisms and those that are unable to grow on artificial culture media [67]. This technique enables the detection and determination of fungal, bacterial, and Oomycete diversity in forest samples, thereby facilitating the development of anticipatory and robust management strategies [67]. The introduction of new tree species, as is happening in the Basque Country as a result of the damage caused by brown spot needle blight in *P. radiata* plantations, may be associated with ecological impacts caused by the introduction of new pathogens or the adaptation of native pathogens to exotic hosts [29]. The first screening using environmental DNA metabarcoding may be helpful in detecting several of these interactions.

In the present study, none of the sampled *Pinus* spp. showed *Dothistroma* species, which are the causal agents of red band needle blight. This result is consistent with a survey conducted in 2020 in the same arboretum, where the pathogen was not detected using conventional PCR or by obtaining isolates from needles of the same *Pinus* spp. [12]. However, damage caused by *Dothistroma* species has been reported in the Basque Country. The previously mentioned survey found that *Dothistroma septosporum* was present in the needles of *P. brutia*, *P. ponderosa*, and *P. nigra* collected from different arboreta. These findings highlight the need to include multiple study areas and larger sample sizes per tree species to obtain a reliable representation of the regional situation. Similarly, *Fusarium circinatum* was not detected in the present study, although this quarantine pathogen was present in wood samples, roots, and asymptomatic herbaceous and shrubby plants in the *P. radiata* plantation that existed prior to the establishment of the arboretum [68].

## 5. Conclusions

In summary, the results demonstrated that alpha diversity indices were higher in asymptomatic needles than in symptomatic needles. The Bray–Curtis dissimilarity index indicated significant differences between symptomatic and asymptomatic needles. Moreover, the trophic mode pathotroph–saprotroph represented the largest proportion in both asymptomatic and symptomatic needles, and the functional guild ‘plant pathogen’ was the most abundant in both symptomatic and asymptomatic needles. The high presence of plant pathogens in symptomless needles may be explained by the presence of latent pathogens that remain dormant until favourable conditions arise. The genus *Lophodermium* showed the highest abundance, particularly in symptomatic needles, and was the only fungal genus that was significantly depleted in asymptomatic needles. Notably, we did not detect the presence of *Dothistroma* species, which is a well-known pathogen in the region. This confirms the need for including additional sampling sites to obtain a reliable characterisation of the fungal communities in the area. Future studies must include a broader range of representative samples per tree species to further refine our understanding of host-driven shifts in fungal communities and strengthen the foundation for sustainable forest management under emerging disease threats.

## Data Availability

This Targeted Locus Study project has been deposited at DDBJ/EMBL/GenBank under the accession KJKG00000000. The version described in this paper is the first version, KJKG01000000. The project metadata is organised under the BioProject identifier PRJNA1393052.
